# The diagnostic and prognostic value of cell division cycle associated gene family in Hepatocellular Carcinoma

**DOI:** 10.7150/jca.46554

**Published:** 2020-07-29

**Authors:** Bowen Wu, Yu Huang, Yingwan Luo, An Ma, Zhaoxing Wu, Yichao Gan, Ying Xu, Rongzhen Xu

**Affiliations:** 1 Department of Hematology (Key Laboratory of Cancer Prevention and Intervention, China National Ministry of Education, Key Laboratory of Molecular Biology in Medical Sciences, Zhejiang Province) and Cancer Institute, The Second Affiliated Hospital, College of Medicine, Zhejiang University, Hangzhou, 310009, China.; 2 Academy of Chinese Medical Sciences, Zhejiang Chinese Medical University, Hangzhou, 310053, China.; 3 Divisions of Pathology and Experimental Hematology and Cancer Biology, Cincinnati Children's Hospital Medical Center, Cincinnati, USA.; 4 Zhejiang Academy of Medical Sciences, Hangzhou 310009, China.; 5 Institute of Hematology, Zhejiang University, Hangzhou, 310009, China.

**Keywords:** cell division cycle, gene family, hepatocellular carcinoma

## Abstract

Cell division cycle associated (CDCA) gene family plays an important role in cells. However, some researchers revealed that overexpression of CDCAs might contribute to the tumor progression in several cancers. Here, we analyzed the role of this gene family in hepatocellular carcinoma (HCC). We used several web tools and found that most of CDCAs were highly expressed in tumor tissues compared to the paracancer tissues in HCC. We then used RT-qPCR to confirm our results. The results showed that CDCA2, CDCA3, CDCA5 and CDCA8 were up-regulated in HCC. We also found that these genes were associated with poor overall survival and relapse free survival except CDCA7. The functional analysis showed that this gene family might take part in many processes, including cell division, apoptosis, DNA damage and DNA repair, which might contribute to the tumor progression. The KEGG pathway analysis showed that these genes participated in several important pathways such as PI3K-Akt signaling pathway and hippo signaling pathway. In conclusion, our findings suggested that CDCA2, CDCA3, CDCA4, CDCA5, and CDCA8 might have potential diagnostic and prognostic values for hepatocellular carcinoma.

## Introduction

Hepatocellular carcinoma (HCC), the most prevalent form of liver cancer, is a massive global problem, with a future increase in incidences predicted. HCC leads to approximately 800,000 deaths every year with a background of severe liver scarring and typically cirrhosis. We are now appreciating that HCC mostly forms after years of chronic liver disease. However, the mechanism of the oncogenesis is largely unknown.

Cell division cycle associated (CDCA) proteins are a group of proteins that play an important role in many biological process such as cell cycle 1-4. Recently some researchers revealed that CDCAs might contribute to the cancer progression. Previous studies had shown that some members of this gene family might be up-regulated in breast cancer, lung adenocarcinoma and pancreatic adenocarcinoma 5-16. And high expression levels of these genes were identified as poorly predictive factors of survival in these cancers 17, 18. Although some researchers suggested that CDCAs might act as oncogenes in hepatocellular carcinoma as well; one of this family, CDCA2, was not revealed clearly so far. Moreover, some CDCAs were identified as oncogenes without validation in clinical samples. Besides, there were no studies analyzed all the six genes together in hepatocellular carcinoma systematically and comprehensively.

In this study, we used several web tools to evaluate the role of CDCAs in hepatocellular cancer. We analyzed the expression of CDCAs in tumor tissues and normal tissues, and then used the RT-qPCR to confirm the results. We also analyzed the relationship between CDCAs and survival. Finally, we predicted the functions of the CDCAs.

## Materials and Methods

### Ethics Statement

This study was approved by the Ethics Committee of the Second Affiliated Hospital, Zhejiang University School of Medicine (Zhejiang, China). Human hepatocellular carcinoma tissues and the paracancer tissues were obtained from Second Affiliated Hospital, School of Medicine, Zhejiang University with their informed consent in accordance with the Declaration of Helsinki. Seven patients diagnosed as HCC by histopathological examination were included in our study. The information of the patients was list in Table S1. All experiments were approved by the ethics committee of Second Affiliated Hospital, School of Medicine, Zhejiang University.

### Oncomine Analysis

The transcriptional levels of CDCAs were analyzed in Oncomine Database (https://www.oncomine.org/resource/login.html). Using a Student's *t* test, the expression levels of CDCAs mRNA were compared between the cancer tissues and the normal samples. The cutoff of *P*-value, fold change and gene rank were 0.01, 1.5 and 10%, respectively.

### GEPIA Analysis

GEPIA (http://gepia.cancer-pku.cn/) is a web server that analyzing the RNA expression based on the data from The Cancer Genome Atlas (TCGA, https://tcga‑data.nci.nih.gov/tcga/) and the Genotype‑Tissue Expression project (GTEx, https://www.gtexportal.org/home/index.html). In this study, we analyzed the expression levels of CDCAs on the Box Plots, using the fold change cutoff of 2 and *P*-value cutoff of 0.01. The TCGA normal data was chosen as the normal control. In the survival analysis, the median of transcripts per kilobase of exon model per million mapped reads (TPM) of each genes were set as the cutoffs to divide the patients into two groups.

### Protein Atlas Analysis

The Human Protein Atlas (https://www.proteinatlas.org/) database is a Swedish-based program aiming to map all the human proteins in cells, tissues and organs using integration of various omics technologies, including antibody-based imaging, mass spectrometry-based proteomics, transcriptomics and systems biology. In this study, the protein levels of the CDCAs were gained from this database.

### cBioPortal Analysis

The cBioPortal for Cancer Genomics (https://www.cbioportal.org/) is a web resource for exploring, visualizing and analyzing multidimensional cancer genomics data. The genomic profiles containing mutations, putative copy number alterations (CNAs) and mRNA expression Z scores (RNA-seq v.2 RSEM) were calculated in this database. Co-expression and network were also conducted in this website based on the online instructions.

### The Database for Annotation, Visualization and Integrated Discovery Analysis

The Database for Annotation, Visualization and Integrated Discovery (DAVID, https://david.ncifcrf.gov/) provides a comprehensive set of functional annotation tools for investigators to understand biological meaning behind large list of genes. We based the Gene Ontology (GO) analysis and the Kyoto Encyclopedia of Genes and Genomes (KEGG) analysis on DAVID website.

### Gene Set Enrichment Analysis

Gene Set Enrichment Analysis (GSEA) was performed by the software GSEA_4.0.2 (https://www.gsea-msigdb.org/gsea/index.jsp). An independent cohort, GSE6764, was used in this analysis. The patients were divided into two groups according to the expression levels of each CDCAs.

### CancerSEA Analysis

CancerSEA (http://biocc.hrbmu.edu.cn/CancerSEA/) is the first dedicated database that aims to comprehensively decode distinct functional states of cancer cells at single-cell resolution. It provides a cancer single-cell functional state atlas, involving 14 functional states of 41,900 cancer single cells from 25 cancer types. It can query which functional states the gene (including PCG and lncRNA) or gene list of interest is related to across different cancer types. It provides PCG/lncRNA repertoires that are highly related to functional states at single-cell resolution. The functions of each CDCAs were predicted in this web tool.

### Real-Time PCR

Total RNA was extracted by using Trizol (Invitrogen) according to the manufacturer's instructions. 500 ng of total RNA was reverse transcribed to cDNA using PrimeScriptTM Ⅱ 1st strand cDNA Synthesis Kit (Takara). RT-qPCR was performed with SYBR® Premix Ex TaqTM II (Tli RNaseH Plus) (Takara) on 7500 Real-Time PCR Systems (Applied Biosystems, USA). Gene expression level was determined by using the ΔΔcycle threshold method normalized to β-Actin. qPCR primers used were as follows: CDCA2 (forward primer ATGACCGGCTGTCTGGAAT and reverse primer GCTGAGACCTTCCTTTCTGGT), CDCA3 (forward GGACCCTGAGACTCCCAGAT and reverse GCCGCTTACCCTGTCGTAG), CDCA4 (forward ATTTGAAACGCTGGAGACT and reverse CCCATCATGCCTGTCAGTA), CDCA5 (forward AGAAAGTCAGGCGTTCCTACAG and reverse GGGAGATTCCAGGGAGAGTCAT), CDCA7 (forward TACAGCCTTCCCGAACTGAC and reverse TAACGAACTGGCCGGTATTT), CDCA8 (forward TTGACTACTTCGCCCTTG and reverse CTTCTTCTTCCTCTTCCACTA) and Actin (forward ACTCTTCCAGCCTTCCTTCC and reverse AGCACTGTGTTGGCGTACAG).

## Results

### CDCA2, CDCA3, CDCA5 and CDCA8 were overexpressed in hepatocellular carcinoma

**Figure 1:**
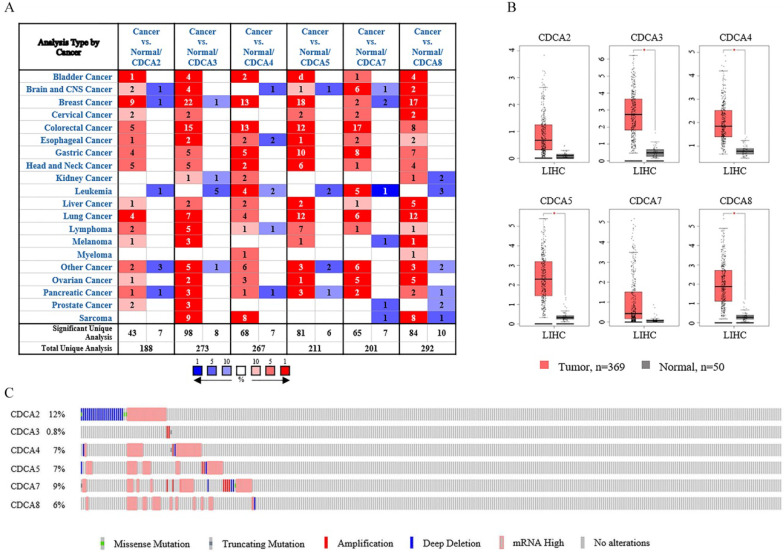
** The transcriptional levels of CDCAs in different types of cancers.** (**A**) The transcriptional levels of CDCAs in different types of cancers (Oncomine). (**B**) The transcriptional levels of CDCAs in hepatocellular carcinoma (GEPIA). (**C**) The expression and mutation patterns of CDCAs in hepatocellular carcinoma (cBioportal).

Six CDCAs have been identified in the human genome, including CDCA2, CDCA3, CDCA4, CDCA5, CDCA7 and CDCA8. On the base of Oncomine database, we compared the expression levels of CDCAs in cancer tissues and in normal samples (Figure 1A). The results showed that the expression levels of CDCAs increased in most of the cancers, but decreased in leukemia.

As the results listed in Table 1, all the CDCAs were highly expressed in liver cancers according to four datasets provided 19-21. In Wurmbach's dataset 20, CDCA2, CDCA3, CDCA4, CDCA5 and CDCA8 were up-regulated in hepatocellular carcinoma (HCC), with fold changes of 1.813, 3.214, 1.832, 2.422 and 1.693, respectively. Roessler's 19 two datasets indicated that CDCD3, CDCA4 and CDCA8 were overexpressed in hepatocellular carcinoma. According to one dataset, the fold changes of these three genes were 1.633 and 1.545 and 1.760, respectively. The other dataset indicated that CDCA8 was significantly up-regulated with a fold change of 1.583. CDCA5, CDCA7 and CDCA8 were highly expressed in Chen's analysis 21. These three genes were up-regulated with fold changes of 4.400, 1.955 and 5.159, respectively in hepatocellular carcinoma. Besides, Chen's dataset also showed that CDCA8 was expressed higher in focal nodular hyperplasia of the liver (fold change=2.194).

To further reveal the transcriptional levels of CDCAs in hepatocellular carcinoma, we next conducted the analysis in Gene Expression Profiling Interactive Analysis (GEPIA). Using the log_2_ (fold change) of 1 and the *P*-value of 0.01, we analyzed 369 hepatocellular carcinoma tissues and 50 normal tissues. As Figure 1B showed, CDCA3, CDCA4, CDCA5 and CDCA8 were up-regulated in hepatocellular carcinoma whereas CDCA2 and CDCA7 exhibited no significant difference. We next calculated the expression patterns of CDCAs in another web tool, cBioPortal. As Figure 1C suggested, this gene family was altered in 25% hepatocellular carcinoma patients (90 cases of total 360 patients). Among all the changes, mRNA up-regulation was the most frequent. Besides, DNA copy number amplification and deep deletion were also observed in the changes.

The dataset of GSE84402 were used to validate the expression of CDCAs. This cohort contained 14 hepatocellular carcinoma tissues and correspondent non-carcinoma tissues. As Figure S1 showed, the five genes, CDCA2, CDCA3, CDCA4, CDCA5 and CDCA8 were all up-regulated in the HCC samples.

**Figure 2:**
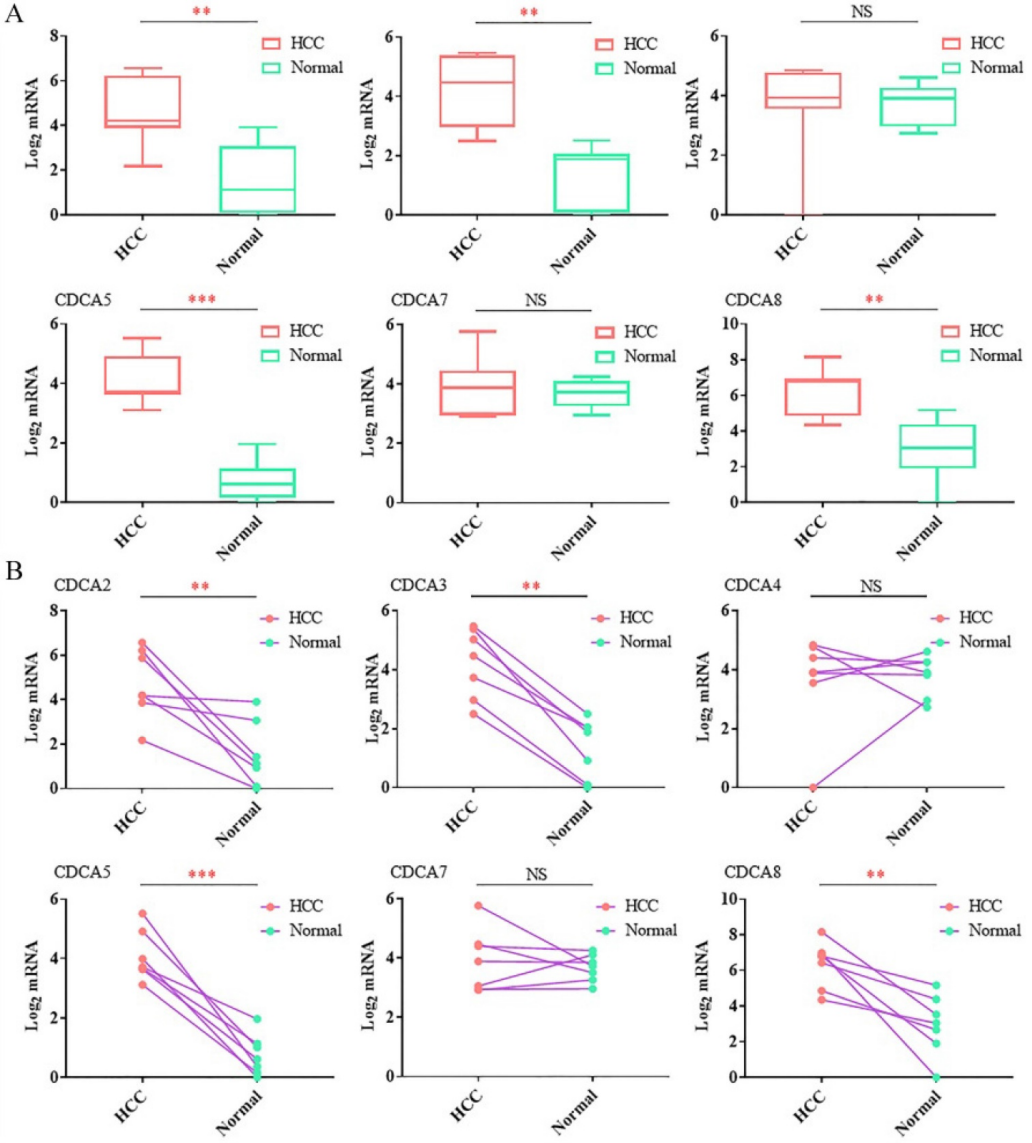
RT-qPCRanalysis of the expression levels of the CDCAs in hepatocellular carcinoma tissues and paracancer tissues.

At last, we used RT-qPCR to confirm the mRNA levels between the tumor tissues and the corresponding paracancer tissues. Seven paired samples were used in RT-qPCR. As Figure 2A showed, the average expression levels of CDCA2, CDCA3, CDCA5 and CDCA8 were significantly up-regulated in tumor tissues compared to the paracancer tissues. After comparing the expression level of the tumor tissue and the paracancer tissue in each case, we found that the expression levels of CDCA2, CDCA3, CDCA5 and CDCA8 were overexpressed in tumor tissues in each patient (Figure 2B). Different from the results in the database, our results showed that there were no significant change of CDCA4 in HCC tissues compared to the normal samples (Figure 2A,B).

**Figure 3:**
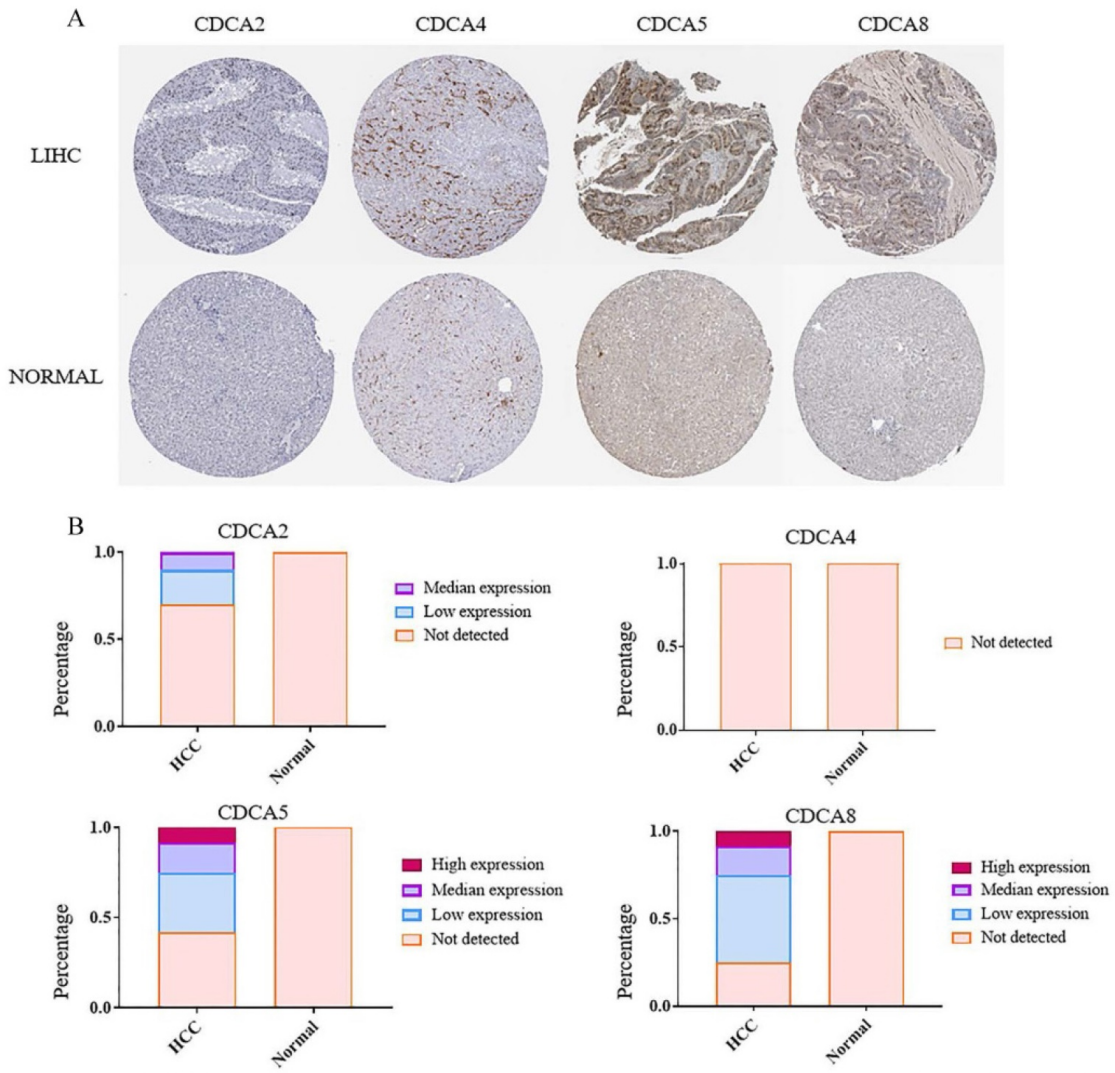
** The protein levels of CDCAs in hepatocellular carcinoma tissues and paracancertissues.** (**A**) The immunohistochemistry of CDCAs in HCC tissues and paracancertissues. (**B**) The protein levels of CDCAs in HCC tissues and paracancer tissues.

The protein evidences of CDCAs were supported by immunohistochemistry (IHC) in The Human Protein Atlas database. Data of four CDCAs were available. The percentages of the patients expressing CDCA2, CDCA5 and CDCA8 were nearly 30%, 60% and 80% (Figure 3B), respectively. And about 30% patients had median or high expression levels of CDCA5 or CDCA8. These three genes, however, could not be detected in the normal liver tissues (Figure 3A, B). The protein of CDCA4 could be detected neither in HCC nor in normal liver tissues. This result of CDCA4 was a support of our RT-qPCT outcome.

### All the CDCAs except CDCA7 might be associated with overall survival and relapse-free survival

**Figure 4:**
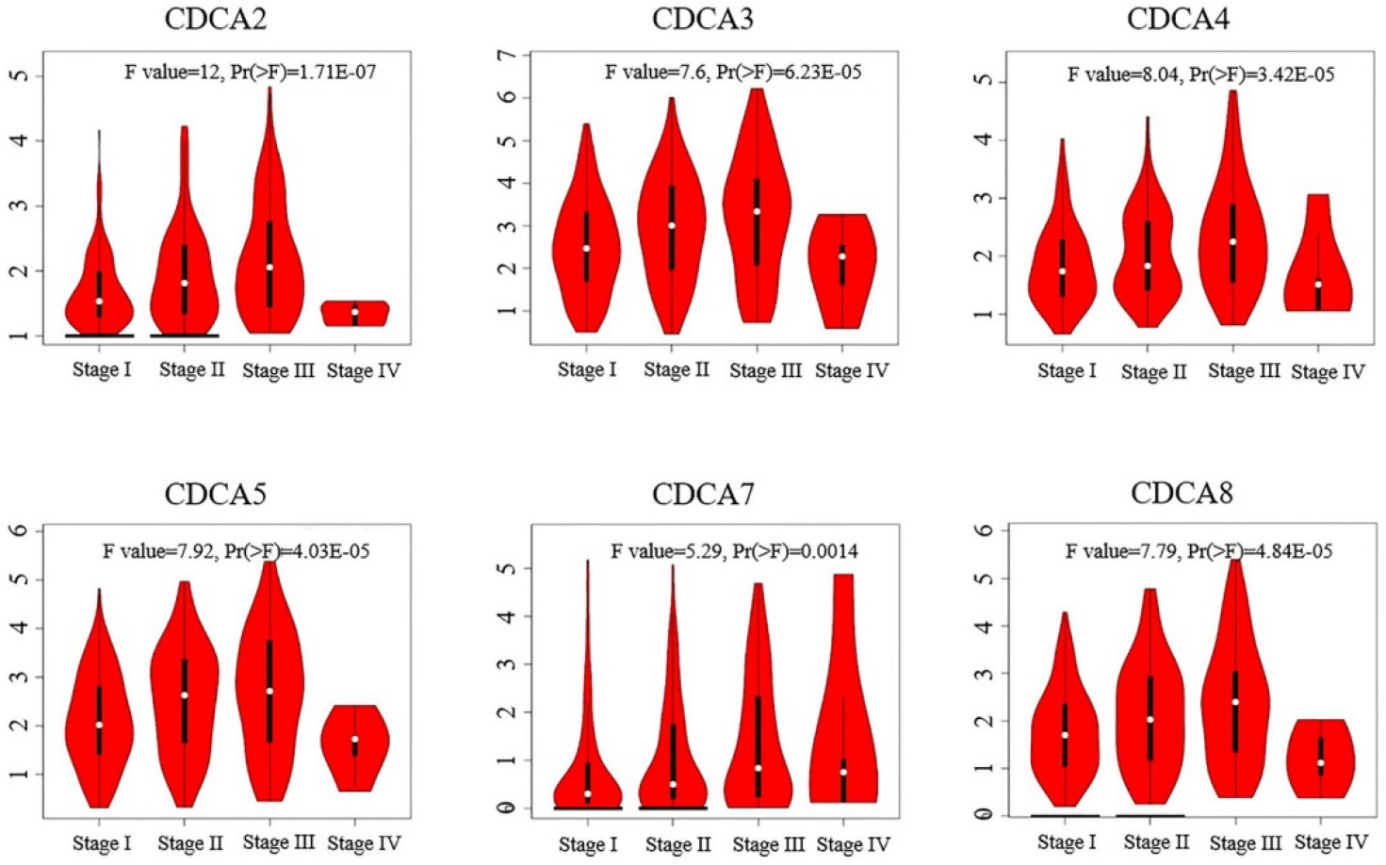
Correlation between expression levels of CDCAs and tumor stages in hepatocellular carcinoma (GEPIA).

We next analyzed the relationships of the expressions of CDCAs with tumor stage of hepatocellular carcinoma using GEPIA. As Figure 4 showed, the expressions of CDCAs significantly varied in different tumor stage. To specify, the mRNA levels of CDCAs were higher in patients classified in stage II than those in stage I. And the median expression level became higher in the patients of stage III, however, lower in the patients of stage IV.

**Figure 5:**
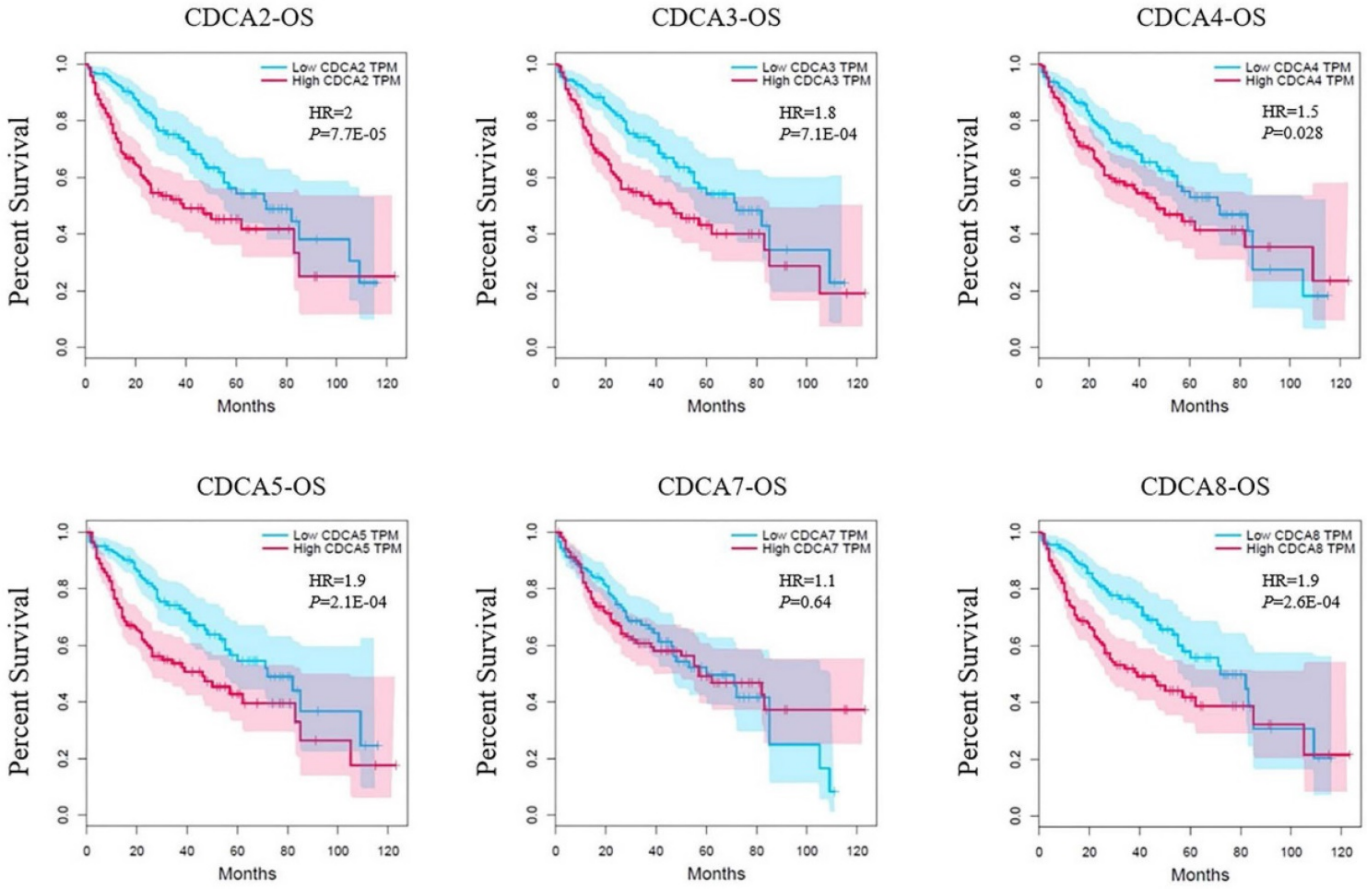
Correlation between CDCAs and overall survival (OS) in hepatocellular carcinoma (GEPIA).

Survival analysis was based on the data in GEPIA as well. Each of the median expression of log_10_ (TPM) of CDCAs was set as the cutoff to divide the patients into high-expressed group and low-expressed group. In our analysis, each CDCA was associated with overall survival (OS) except for CDCA7 (Figure 5). The CDCA2 had the highest hazard ratio (HR=2, *P*=7.7E-06) that indicated an increased risk of the patients in high-expressed group. Similarly, patients who had high expression levels of CDCA3 (HR=1.8, *P*=7.1E-04), CDCA4 (HR=1.6, *P*=0.028), CDCA5 (HR=1.9, *P*=2.1E-04) or CDCA8 (HR=1.9, *P*=2.6E-04) might be related to worse overall survival as well.

**Figure 6:**
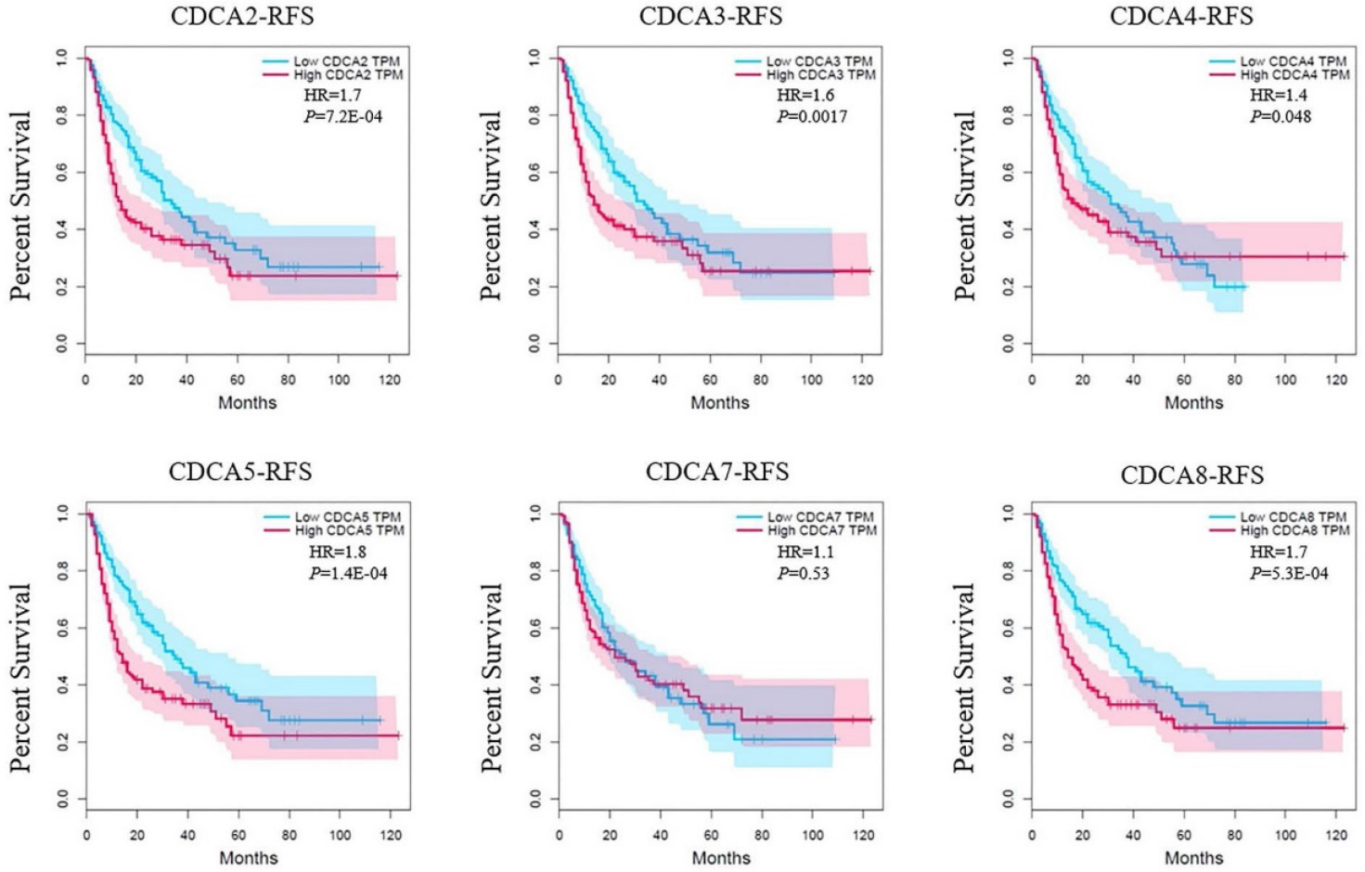
Correlation between CDCAs and relapse free survival (RFS) in hepatocellular carcinoma (GEPIA).

The results of relapse free survival (RFS) were similar. The CDCA5 had a HR of 1.8 (*P*=1.4E-04) that ranked the top (Figure 6). High expression levels of CDCA2 (HR=1.7, *P*=7.2E-04), CDCA3 (HR=1.6, *P*=0.0017), CDCA4 (HR=1.4, *P*=0.048) or CDCA8 (HR=1.7, *P*=5.3E-04) suggested poor disease free survival as well.

As the data in GEPIA were based on the TCGA database, we then validated the correlations of CDCAs and survival in GEO database. The words of “hepatocellular” were used as the search term. The expression profiling by MPSS, RT-PCR, SAGE, SNP, array genome tiling array and high throughput sequencing were chosen in the “Study type” column in the database. The studies that contained sufficient cases, data of survival time and data of expression levels of all the CDCAs were involved into the next calculation. After analyzed, the dataset of GSE116174 that contained overall survival time was included. As Figure S2, S3 showed, high expression levels of CDCA2, CDCA3, CDCA4, CDCA5 and CDCA8 were associated with poor OS.

### Predictions of the functions and pathways of CDCAs in hepatocellular carcinoma

**Figure 7:**
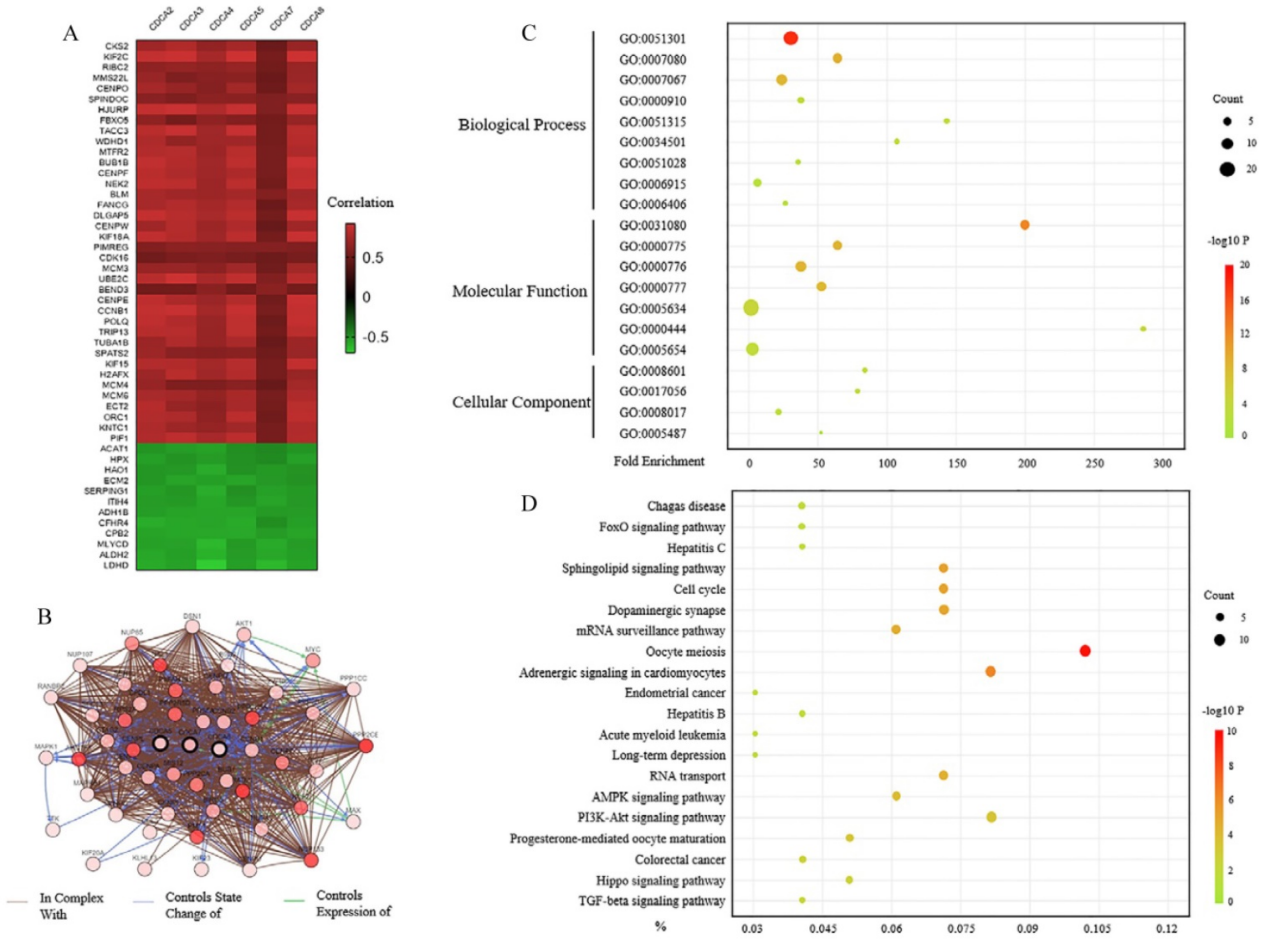
** The predictive functions of CDCAs in hepatocellular carcinoma.** (**A**) Genes that were correlated with CDCAs alterations (cBioportal). (**B**) The network for CDCAs and their neighborgenes (cBioportal). (**C,D**) GOenrichment (C) and KEGG (D) analysis of CDCAs (DAVID).

To discover the genes that significantly changed with CDCAs alteration, we analyzed the data with cBioPortal. In Figure 7A, we listed 50 genes that correlated with all the CDCAs in mRNA level. Among them, 38 genes were up-regulated and 12 genes were down-regulated. The protein-protein interaction network was also performed using this web tool. As Figure 7B showed, 50 neighbor genes significantly interacted with CDCAs, such as CCNB1, CCNB2, MYC, MAX and AKT1. These closely associated genes were involved together in the next analysis.

The functions of CDCAs and the genes significantly correlated with CDCAs were predicted by analyzing Gene Ontology (GO) and the Kyoto Encyclopedia of Genes and Genomes (KEGG) in the Database for Annotation, Visualization and Integrated Discovery (DAVID). GO enrichment analysis predicted the functional roles via three aspects, biological processes, cellular components and molecular functions respectively. We found that the CDCAs mainly participated in the processes of cell division (GO:0051301), mitotic metaphase plate congression (GO:0007080), mitotic nuclear division (GO:0007067), cytokinesis (GO:0000910), mRNA transport (GO: 0051028), protein localization to kinetochore (GO:0034501) and mRNA export from nucleus (GO: 0006406) (Figure 7C). Besides, we discovered that CDCAs might be involved in the apoptotic process (GO:0006915).

We then investigated the pathways related to the CDCAs using KEGG analysis. The results showed that FoxO signaling pathway (bta04068), Cell cycle (bta04110), AMPK signaling pathway (bta04152), PI3K-Akt signaling pathway (bta04151), Hippo signaling pathway (bta04390) and TGF-beta signaling pathway (bta04350) had correlations with CDCAs (Figure 7D).

**Figure 8:**
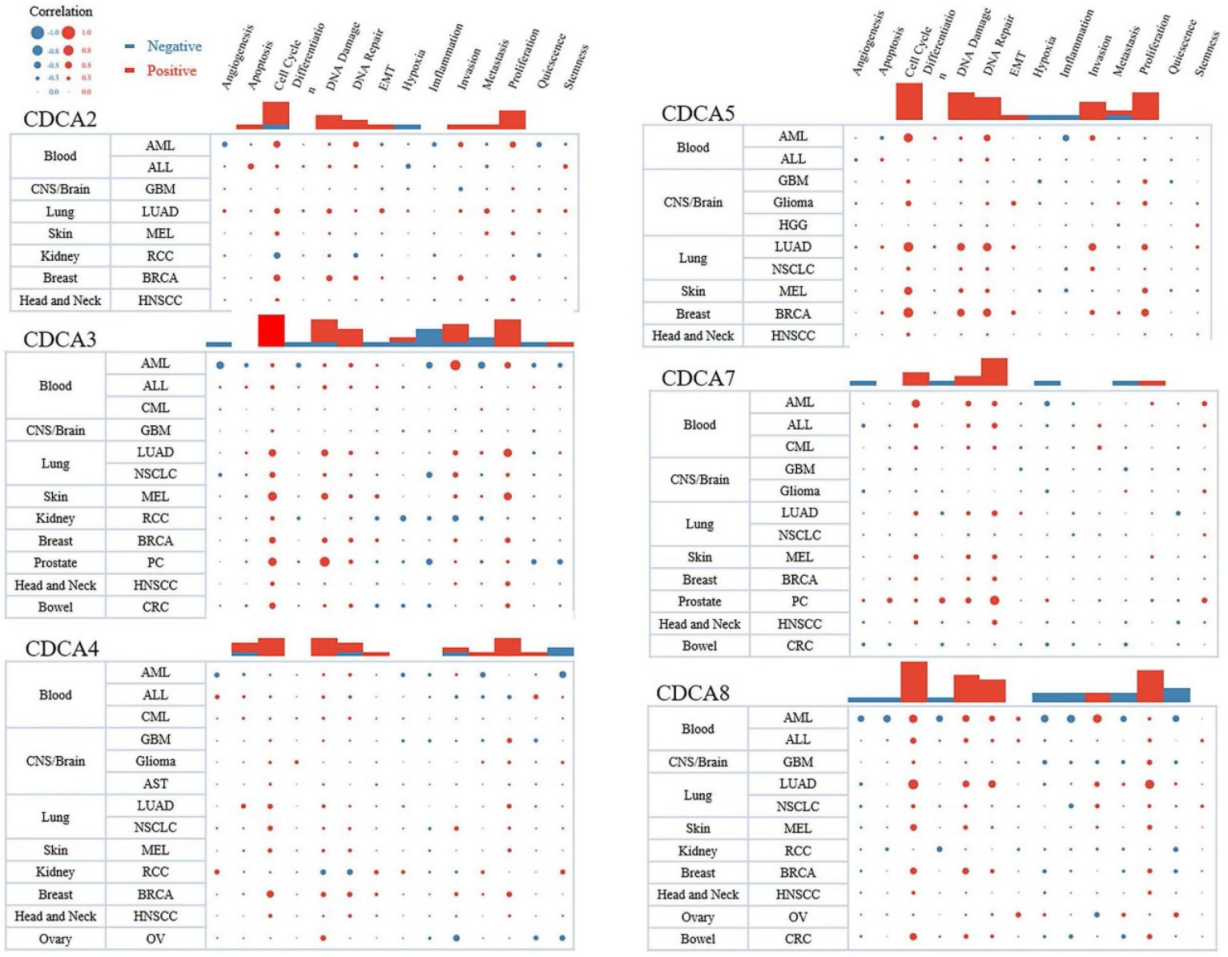
The predictive functions of each CDCA in different types of cancers (CanserSEA).

Using the web tool of CancerSEA, we demonstrated the pan-cancer analysis of the function of each CDCA. We predicted the function from 14 aspects, such as cell cycle, apoptosis, DNA repair, invasion and inflammation. The sizes of dots represented the strengths of the associations and the colors indicated positive or negative correlations. As Figure 8 showed, all the CDCAs had positive correlations with cell cycle and proliferation in most cancers, especially CDCA2, CDCA3, CDCA5 and CDCA8. The results were consistent with our functional prediction and strongly indicated that CDCAs might play an important role in the proliferation of hepatocellular carcinoma cells. Moreover, these four genes were also associated with DNA damage and DNA repair. Three of them were associated with invasion, respectively CDCA3, CDCA5 and CDCA8. It was worth noting that CDCA3 negatively regulated the inflammation in several types of cancers.

To validate the function of CDCAs, we then download the data of GSE7606 and analyzed the function of CDCAs using GSEA. A total of 75 patients were involved in this dataset. According to the median expression level of each CDCA gene, the patients were divided into two groups. Both of the CDCA-High group and CDCA-Low group contained 37 patients. Among the functions that predicted by DAVID and CancerSEA above, 7 GO terms could be find in the results of GSEA. These biological processes were cytokinesis, mitotic metaphase plate congression, mitotic nuclear division, mRNA export from nucleus, mRNA transport, protein localization to kinetochore and DNA repair. The results of GSEA showed that CDCA4 and CDCA5 participated in all the seven processes (Figure S6, S7). The CDCA2 took part in six processes except of mitotic metaphase plate congression (Figure S4). Similarly, CDCA8 was associated in six processes except of mRNA transport (Figure S8). CDCA3 took part in the processes of cytokinesis, mitotic metaphase plate congression, mitotic nuclear division, protein localization to kinetochore and DNA repair (Figure S5). In conclusion, all the results above strongly indicated that the CDCA family might play an important role in the processes of cell division, DNA damage and repair, and moreover, the process of apoptosis.

## Discussion

In this study, we analyzed the diagnostic and prognostic value of CDCA gene family in hepatocellular carcinoma. We compared the expression levels of the mRNA and protein in tumor tissues and normal tissue. We found that CDCA2, CDCA3, CDCA5 and CDCA8 were up-regulated in HCC. By analyzing the survival, we noticed that high expression levels of CDCA2, CDCA3, CDCA4, CDCA5 and CDCA8 might be related to the poor survival. We also predicted that this family played an important role in cell division by participating in multiple biological processes and pathways.

Previous studies had revealed that CDCA2 might be an oncogene in some cancers, such as luminal breast cancer 17, lung adenocarcinoma 18 and pancreatic ductal adenocarcinoma. The expression level of CDCA2 was up-regulated in breast cancer as well as in lung cancer. And the patients with highly CDCA2 expressed had shorter survival. However, the role of CDCA2 in hepatocellular carcinoma remained unknown. Our study demonstrated a similar CDCA2 expression pattern in hepatocellular carcinoma. High expression level of CDCA2 was related to a poor OS and RFS. The experiments in cell line indicated that CDCA2 promoted the growth of A549, H1299 18, SW480, DLD-1 5 and OSCC 6 cells via motivating the cells from G0/G1 phase into the S phase. Besides, knockdown of CDCA2 might induce apoptotic cell death 6. The results were consistent with our data in functional prediction.

Little is known about the expression and the role of CDCA4 in cancer until now. Xu et al 10 found that CDCA4 was a downstream gene of the Nrf2 signaling pathway regulating cell proliferation and apoptosis of breast cancer cell line. Others pointed out that it played a role in cell proliferation via improving the level of E2F 11. Recently a study suggested that CDCA4 was highly expressed in HCC and correlated with poor survival. Our results supported the conclusion that the mRNA level of CDCA4 was associated with poor survival. However, the expression of this gene remained unchanged between the HCC patients and the normal samples in our results of real-time PCR. Consistently, the results in Protein Atlas database showed that the protein level of CDCA4 could be detected neither in HCC patients nor in normal samples. These results supported our data of RT-qPCR. Our results suggested that CDCA4 could be a prognostic biomarker, however, might not be a diagnostic biomarker in HCC. A larger sample was needed to confirm this conclusion.

CDCA8 was also considered as an oncogene 22 in several types of cancers such as breast cancer 14, bladder cancer 23 and cutaneous melanoma 24. The resent two studies and we all indicated that the mRNA level of CDCA8 was up-regulated in HCC and was associated with poor survival. However, there were no validations in the previous studies. In our paper, we used several web tools to show that CDCA8 was highly expressed in HCC patients either in mRNA level or in protein level. And then we validated the result of RNA level with RT-qPCR. Our results were supports and supplements to the previous studies.

CDCA3 and CDCA5 had been considered as potential oncogenes in hepatocellular carcinoma. Some bioinformatics analysis had suggested that CDCA3 contributed to the pathological process in several cancers, including breast cancer 25, bladder cancer 23 and liver cancer 26, 27. Previous studies had shown that the functional loss of CDCA3 might block the transition from the GO/G1 phase of BGC823, H1 and Sa3 cells 2, 7-9. Three retrospectively cohort studies had indicated that CDCA5 was overexpressed in HCC 28-30. The three studies had shown that high expression level of CDCA5 had correlation with III histological grade, III-IV TNM stage, multiple tumor number and poor OS. Our results were coincident with the previous studies.

In conclusion, we supposed that CDCA2, CDCA3, CDCA4, CDCA5 and CDCA8 might be oncogenes in hepatocellular carcinoma. We ensured that CDCA2, CDCA3, CDCA5 and CDCA8 were highly expressed in HCC. And high expression levels of CDCA2, CDCA3, CDCA4, CDCA5 and CDCA8 might reduce the survival time of patients. We predicted that this gene family took part in cell cycle, DNA repair, invasion, proliferation and other processes in hepatocellular carcinoma. However, further validation work should be done to support our hypothesis.

## Supplementary Material

Supplementary figures and tables.

## Figures and Tables

**Table 1:** The significant changes of CDCAs expressions in transcriptional level between hepatocellular carcinoma tissues and normal liver tissues

Gene	Type of Gastric Cancer vs Normal	Fold Change	*P* Value	t test	Reference
CDCA2	Hepatocellular Carcinoma	1.813	1.94E-04	3.877	Wurmbach
CDCA3	Hepatocellular Carcinoma	3.241	3.39E-08	6.686	Wurmbach
	Hepatocellular Carcinoma	1.633	6.40E-42	16.083	Roessler
CDCA4	Hepatocellular Carcinoma	1.832	1.55E-05	4.765	Wurmbach
	Hepatocellular Carcinoma	1.545	8.68E-38	14.390	Roessler
CDCA5	Hepatocellular Carcinoma	4.400	4.55E-24	11.810	Chen
	Hepatocellular Carcinoma	2.422	4.86E-06	5.128	Wurmbach
CDCA7	Hepatocellular Carcinoma	1.955	7.28E-08	5.512	Chen
CDCA8	Hepatocellular Carcinoma	5.159	3.98E-24	12.080	Chen
	Focal Nodular Hyperplasia of the Liver	2.194	3.98E-10	7.291	Chen
	Hepatocellular Carcinoma	1.760	1.66E-06	6.008	Roessler
	Hepatocellular Carcinoma	1.693	2.19E-05	4.676	Wurmbach
	Hepatocellular Carcinoma	1.583	1.99E-37	14.357	Roessler 2
