# Supplementary material for: The diagnostic and prognostic value of cell division cycle associated gene family in Hepatocellular Carcinoma
Source: J Cancer. 2020 Jul 29;11(19):5727–37. doi: 10.7150/jca.46554 (PMC7477449; doi:10.7150/jca.46554)
Supplement: Supplementary file 1 — Supplementary figures and tables. [file jcav11p5727s1.pdf]

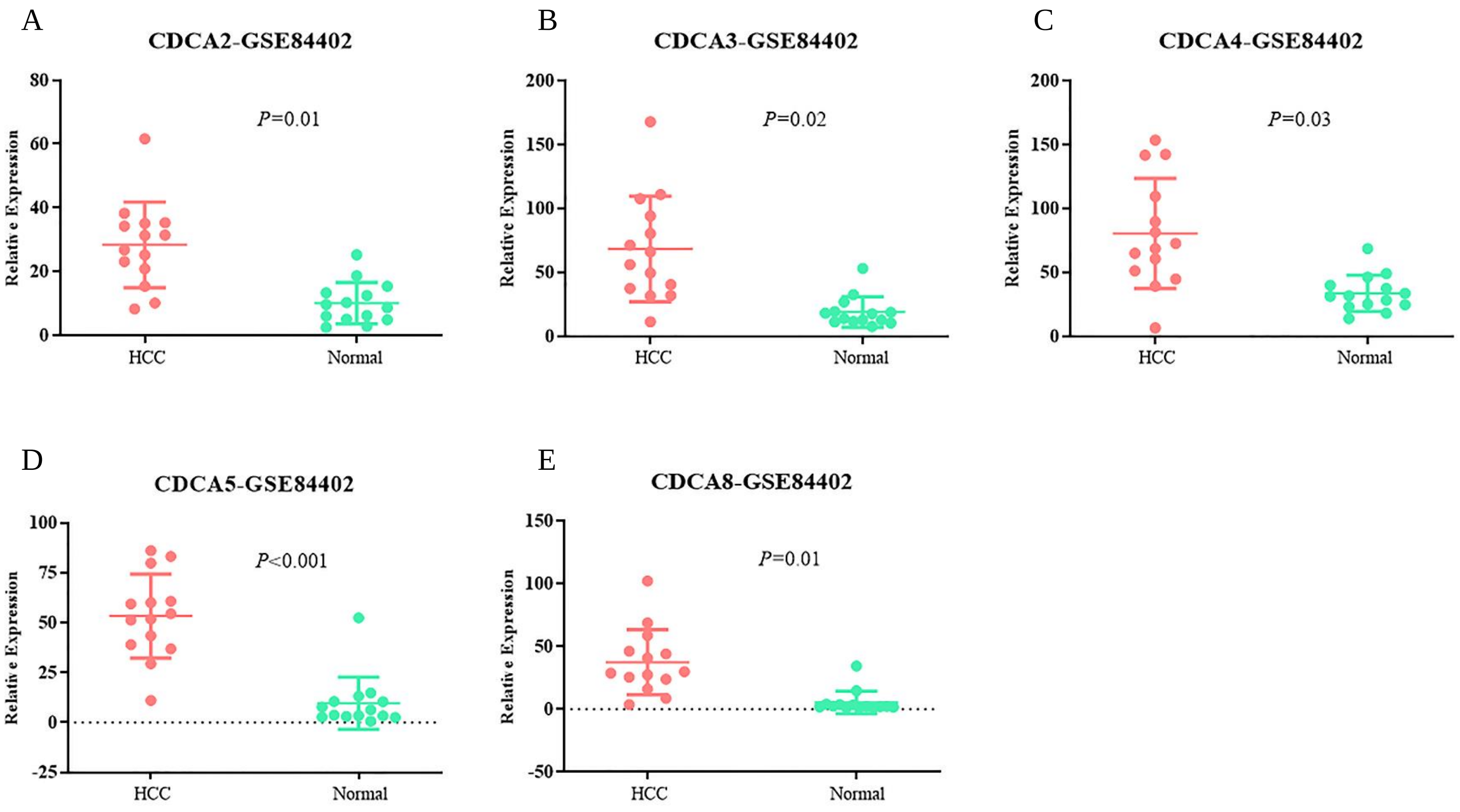

Figure S1. Validation of the expression levels of CDCAs in HCC in GSE84402. (A-E) CDCA2 (A), CDCA3 (B), CDCA4 (C), CDCA5 (D) and CDCA8 (E) were up-regulated in hepatocellular carcinoma.

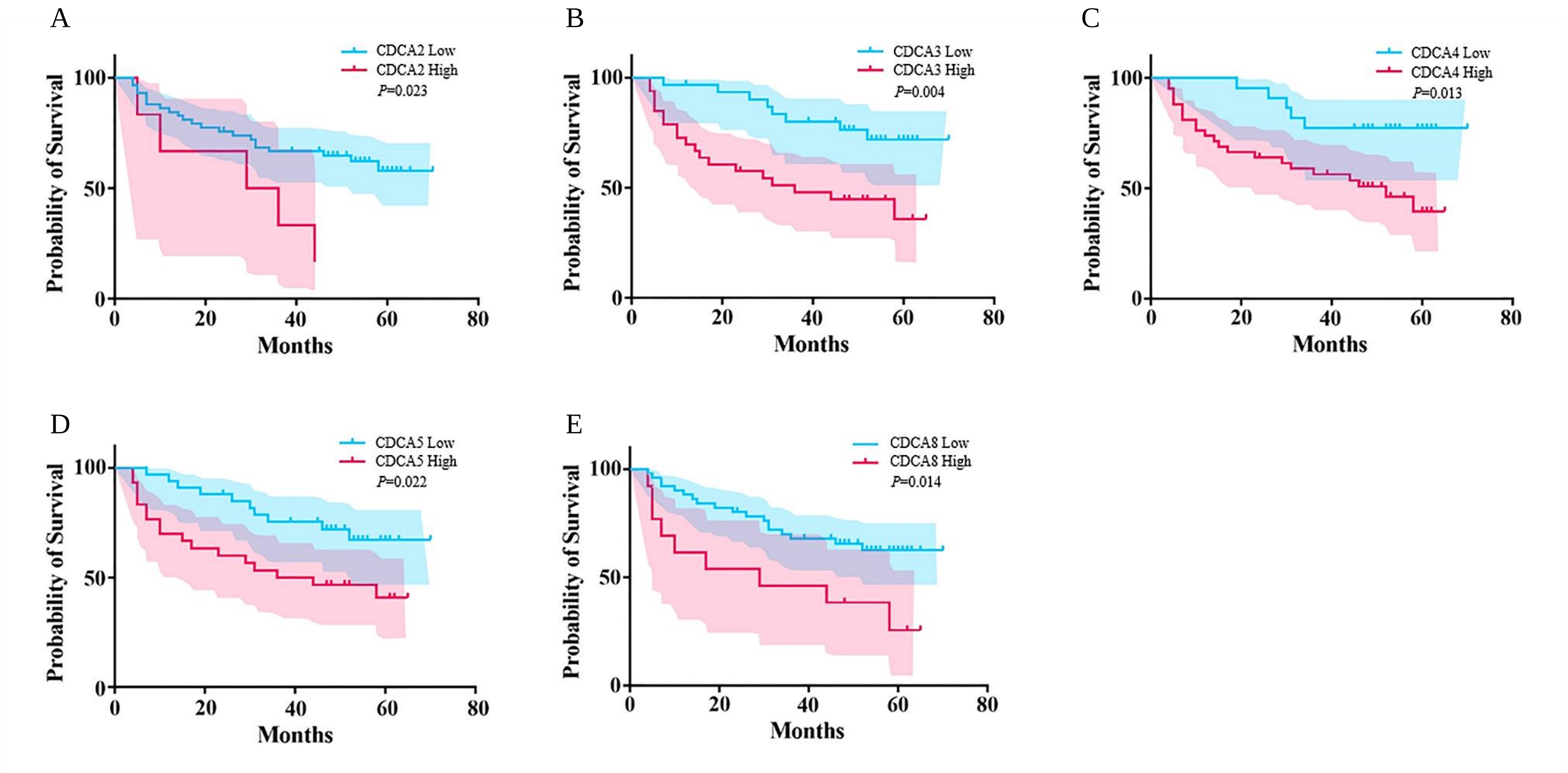

Figure S2. Validation of the correlation between CDCAs and overall survival in GSE116174. (A-E) High expression levels of CDCA2 (A), CDCA3 (B), CDCA4 (C), CDCA5 (D) and CDCA8 (E) were associated with poor OS in hepatocellular carcinoma.

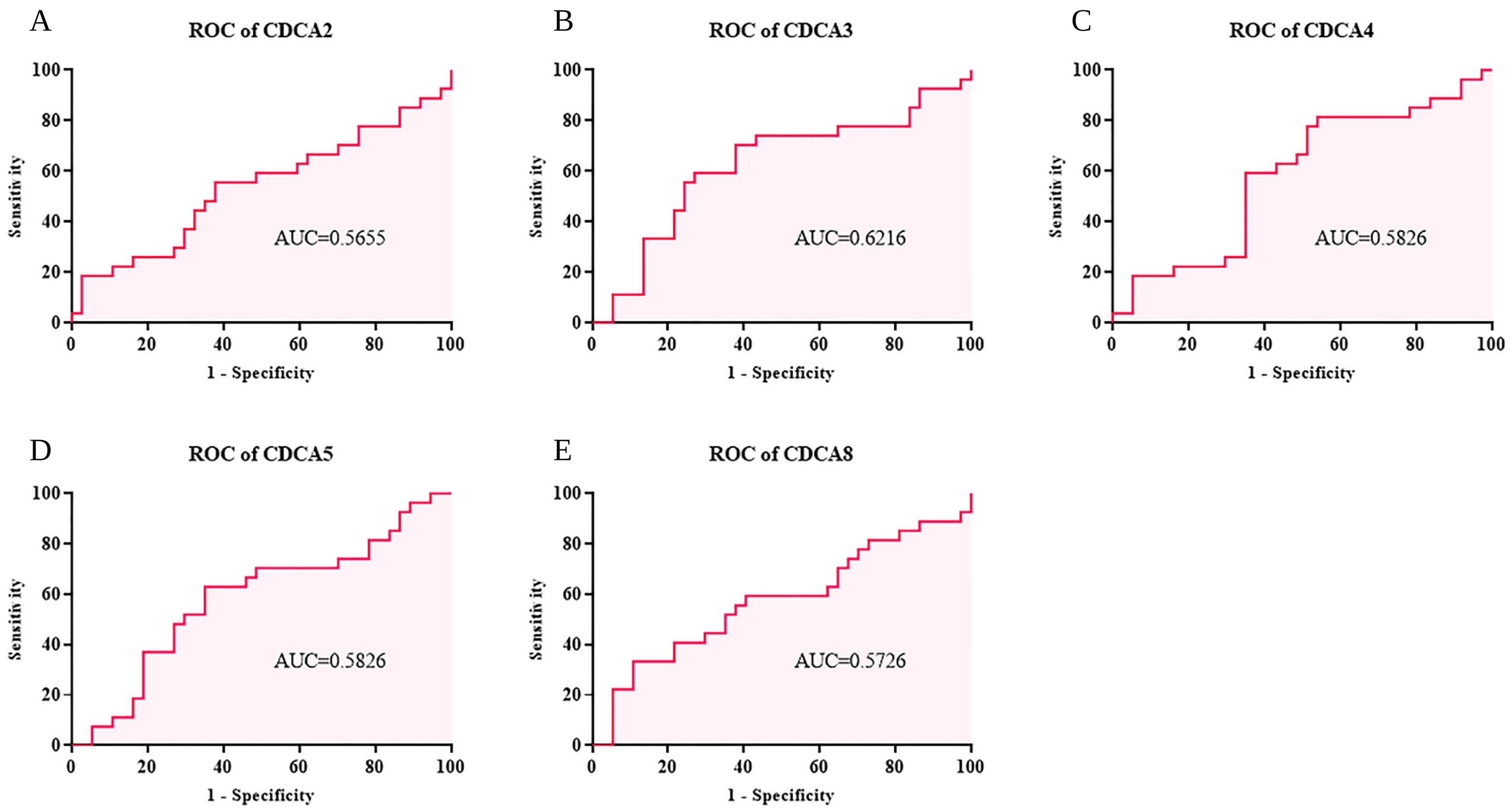

Figure S3. ROC curves of CDCAs in GSE116174

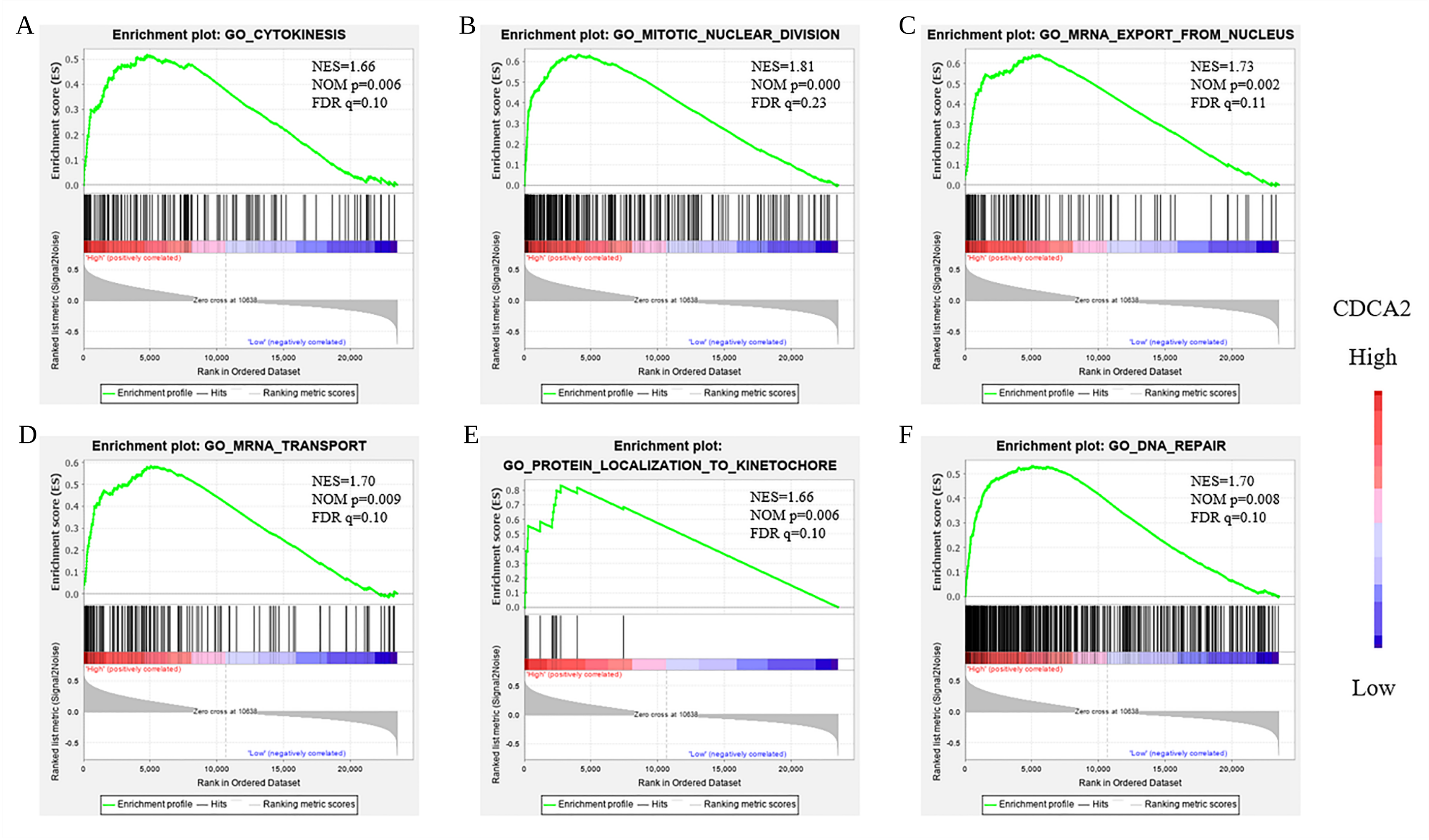

Figure S4. Validation of the main functions of CDCA2 in GSE6764. (A-F) High expression level of CDCA2 might promote the biological processes of cytokinesis (A), mitotic nuclear division (B), mRNA export from nucleus (C), mRNA transport (D), protein localization to kinetoshore (E) and DNA repair (F).

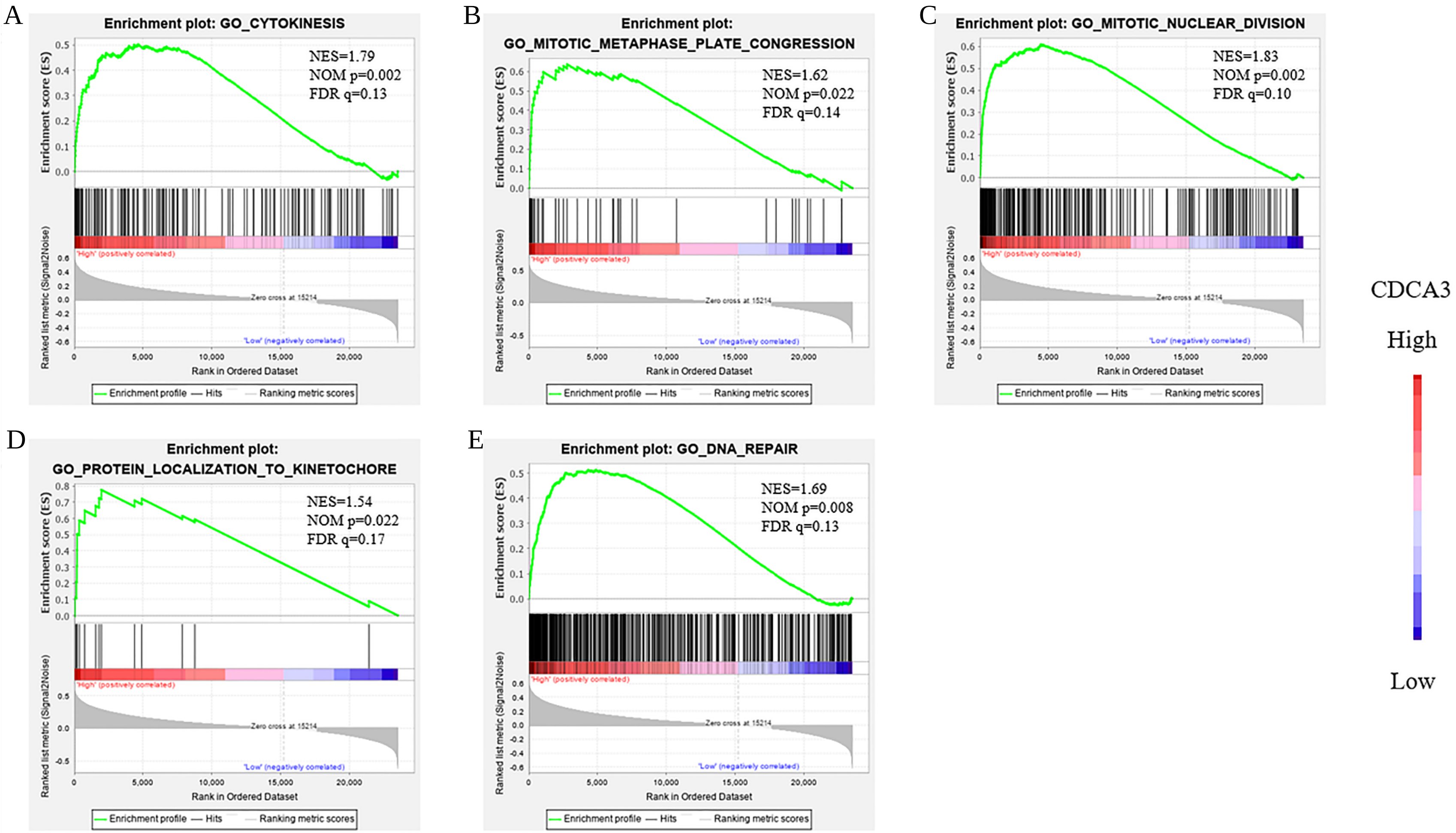

Figure S5. Validation of the main functions of CDCA3 in GSE6764. (A-E) High expression level of CDCA3 promoted the biological processes of cytokinesis (A), mitotic metaphase plate congression (B), mitotic nuclear division (C), protein localization to kinetoshore (D) and DNA repair (E).

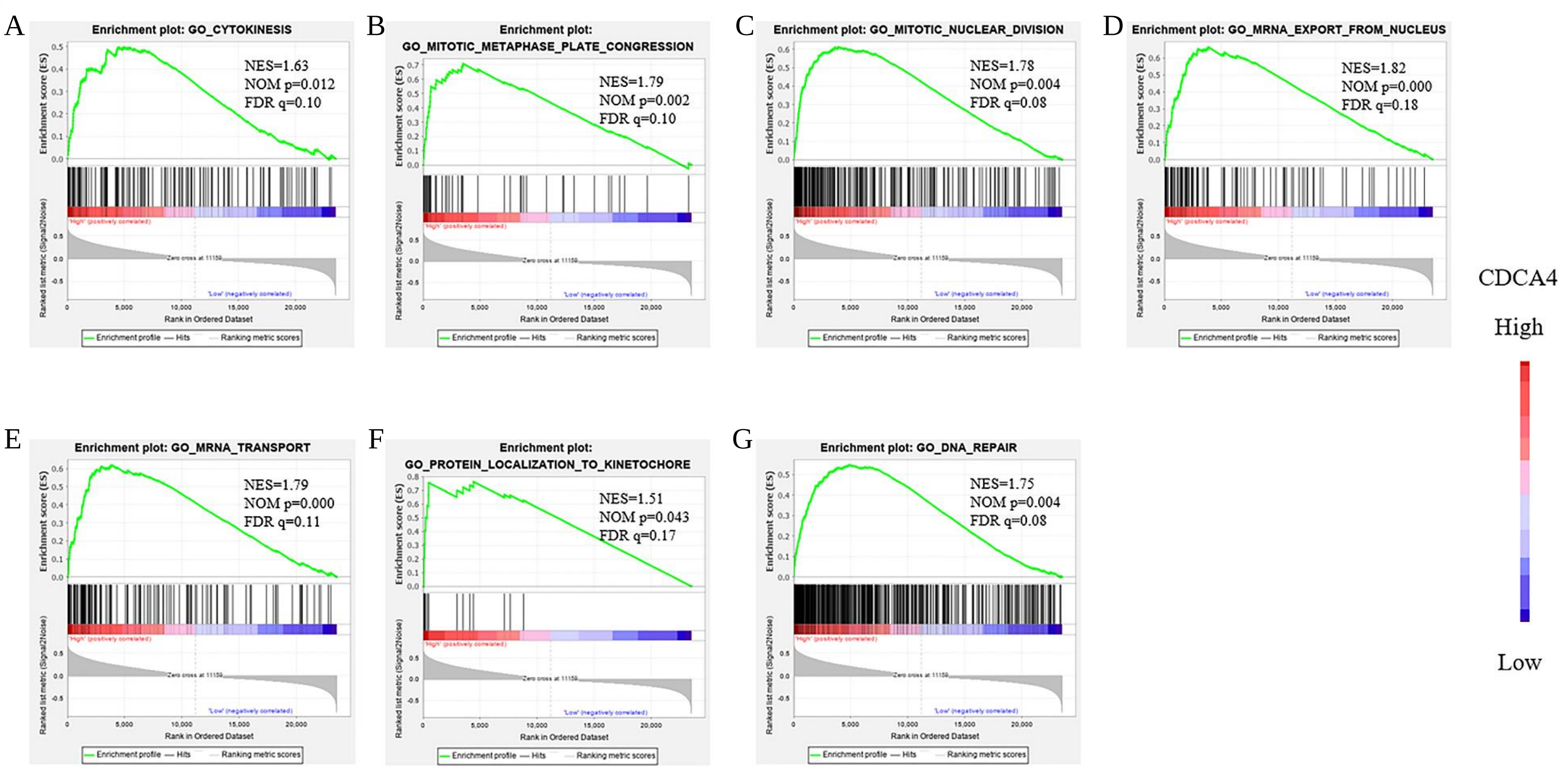

Figure S6. Validation of the main functions of CDCA4 in GSE6764. (A-G) High expression level of CDCA4 promoted the biological processes of cytokinesis (A), mitotic metaphase plate congression (B), mitotic nuclear division (C), mRNA export from nucleus (D), mRNA transport (E), protein localization to kinetoshore (F) and DNA repair (G).

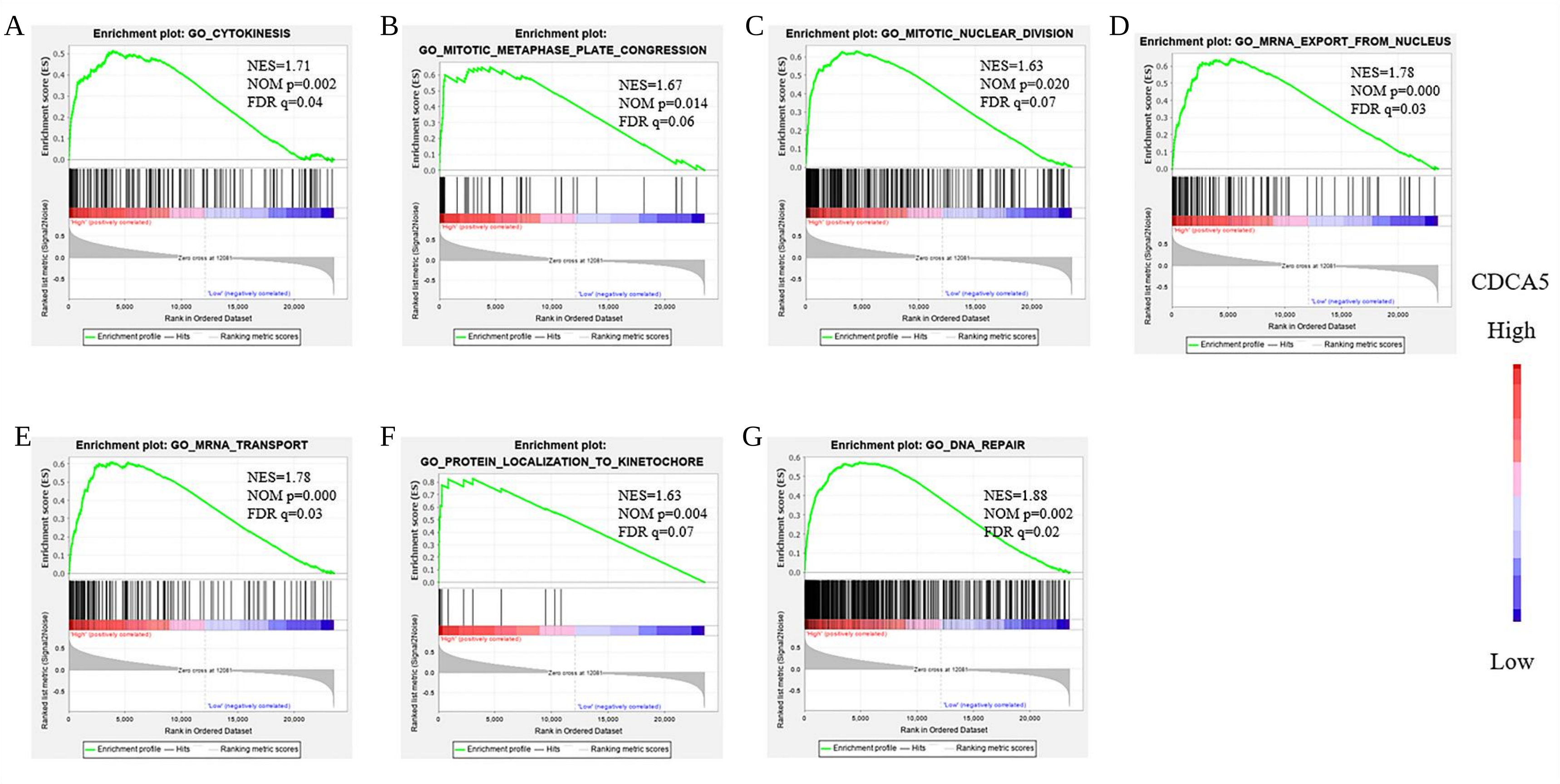

Figure S7. Validation of the main functions of CDCA5 in GSE6764. (A-F) High expression level of CDCA5 promoted the biological processes of cytokinesis (A), mitotic metaphase plate congression (B), mitotic nuclear division (C), mRNA export from nucleus (D), mRNA transport (E), protein localization to kinetoshore (F) and DNA repair (G).

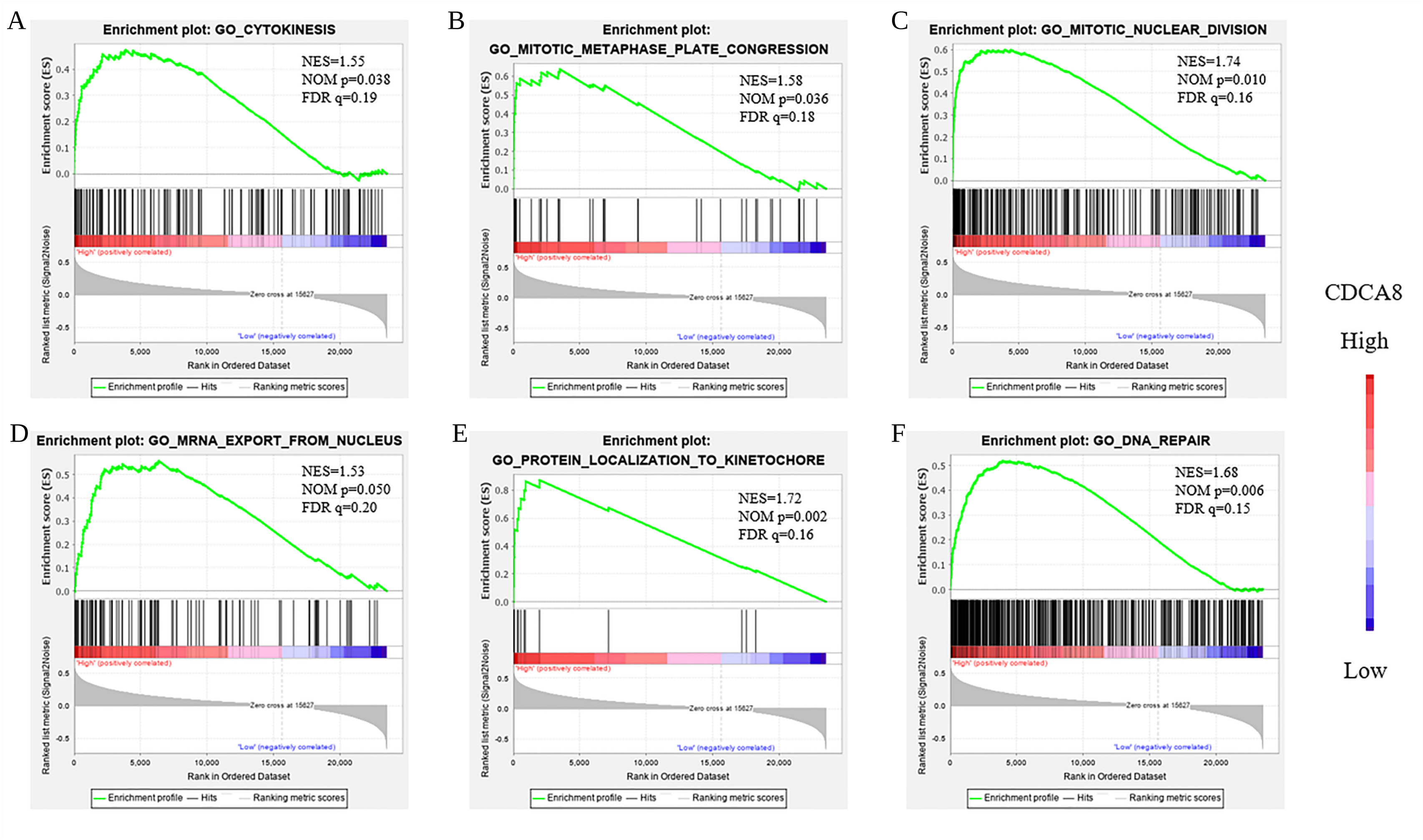

Figure S8. Validation of the main functions of CDCA8 in GSE6764. (A-E) High expression level of CDCA8 promoted the biological processes of cytokinesis (A), mitotic metaphase plate congression (B), mitotic nuclear division (C), mRNA export from nucleus (D), protein localization to kinetoshore (E) and DNA repair (F).

Table S1. Clinical data of the patients that used in the RT-qPCR

	Age	Gender	HBV	Alcohol
HCC1	51	M	Yes	No
Paired Paracancer1				
HCC2	43	M	Yes	No
Paired Paracancer2				
HCC3	65	M	No	No
Paired Paracancer3				
HCC4	58	M	Yes	No
Paired Paracancer4				
HCC5	52	F	Yes	Yes
Paired Paracancer5				
HCC6	55	M	Yes	No
Paired Paracancer6				
HCC7	59	M	Yes	No
Paired Paracancer7				
